# Toxicological response of the model fungus *Saccharomyces cerevisiae* to different concentrations of commercial graphene nanoplatelets

**DOI:** 10.1038/s41598-020-60101-7

**Published:** 2020-02-24

**Authors:** Maria Suarez-Diez, Santiago Porras, Felix Laguna-Teno, Peter J. Schaap, Juan A. Tamayo-Ramos

**Affiliations:** 1 Laboratory of Systems and Synthetic Biology, Wageningen University & Research, Stippeneg, 4 6708WE Wageningen, The Netherlands; 2 Departamento de Economía Aplicada, University of Burgos, Plaza Infanta Doña Elena, s/n, 09001 Burgos, Spain; 3 International Research Centre in Critical Raw Materials-ICCRAM, University of Burgos, Plaza Misael Bañuelos s/n, 09001 Burgos, Spain

**Keywords:** Nanotoxicology, Fungi, Transcriptomics

## Abstract

Graphene nanomaterials have attracted a great interest during the last years for different applications, but their possible impact on different biological systems remains unclear. Here, an assessment to understand the toxicity of commercial polycarboxylate functionalized graphene nanoplatelets (GN) on the unicellular fungal model *Saccharomyces cerevisiae* was performed. While cell proliferation was not negatively affected even in the presence of 800 mg L^−1^ of the nanomaterial for 24 hours, oxidative stress was induced at a lower concentration (160 mg L^−1^), after short exposure periods (2 and 4 hours). No DNA damage was observed under a comet assay analysis under the studied conditions. In addition, to pinpoint the molecular mechanisms behind the early oxidative damage induced by GN and to identify possible toxicity pathways, the transcriptome of *S. cerevisiae* exposed to 160 and 800 mg L^−1^ of GN was studied. Both GN concentrations induced expression changes in a common group of genes (337), many of them related to the fungal response to reduce the nanoparticles toxicity and to maintain cell homeostasis. Also, a high number of genes were only differentially expressed in the GN800 condition (3254), indicating that high GN concentrations can induce severe changes in the physiological state of the yeast.

## Introduction

Graphene and derived nanomaterials (GFNs) are of great interest for different industrial applications, and currently hundreds of companies produce commercial GFNs worldwide^1,2^. In the same line, many research programs have been launched at global scale, aiming to achieve foundational breakthroughs in the generation of scientific knowledge and the development of new technologies around these 2D carbon derived nanomaterials. For example, the European Union (EU) started the Graphene Flagship research program in 2013, with a total budget of 1 billion Euros, being one of the largest research initiatives ever deployed in Europe.

Given the impact that new developments based on graphene are expected to have on future industry worldwide, it is necessary that any possible unwanted societal impacts and risks related to them are determined. Considering the increasing applications, there is a growing likelihood of GFNs release into the environment, which could lead to human and ecosystem exposure with potentially harmful effects. For this reason, the identification of possible safety issues related to the generation, utilisation and disposal of graphene-based materials is essential. Toxicological analyses are also necessary in view of their possible biomedical and biotechnological applications^3,4^. Attending to the morphological and physical properties of this carbon derived nanomaterial, the potential risks to the health of animals, humans and the environment are clear^5^. Most of the studies focusing on graphene biological applications, nanosafety, and in the determination of underlying toxicity mechanisms have been done on mammal cell lines and laboratory animals^4,6,7^. These studies have been essential to obtain insights on how GFNs interact with biological systems and biomolecules for different applications, and to understand factors determining their toxicity, which have been found to be different depending on the animals or cell models used, the administration routes, or the physicochemical properties of the selected nanomaterials. In these studies, several typical mechanisms underlying GFN toxicity have been revealed, for instance, physical destruction, induction of oxidative stress, DNA damage, inflammatory response, apoptosis, autophagy, and necrosis^6–8^.

In relation to studies investigating the biological impact of GFNs on microbial systems, many publications have reported the antibacterial properties of different graphene derivatives and composites^9–13^. In case of the interactions between fungi and graphene-based materials, most of the efforts have focused on improving the antifungal properties of GFNs through their modification with antimycotic drugs, peptides or metals^14–17^. Also, applications involving the fungus *Saccharomyces cerevisiae* and graphene derivatives have been investigated by interfacing graphene oxide and yeast cells^18–20^, aiming for future applications where the cellular physiology can be integrated with electrical read outs^21^, and for the development of environmentally friendly-cost effective methodologies for the preparation of surface modified graphene^19^. However, very little is known about the specific fungal response to the presence of graphene in the environment, such as possible physiological changes or the induction of toxicity pathways.

Determining the eliciting factors of nanoparticles toxicity in a certain microbial system requires a joint physicochemical and biological approach^22^. Therefore, in this work we combined a thorough characterization of a commercial sample of polycarboxylate functionalized graphene nanoplatelets (GN), with a set of physiological analyses in *S. cerevisiae* and the study of the microorganism global transcriptional response to the presence of the nanomaterial. The introduction of polycarboxylate groups in graphitic structures increases the hydrophilicity of the surface and the dispersibility of the nanomaterial in polar solvents^23^, which could enhance the contact between the nanomaterial and the fungal cell, that being the reason for the choice of GN in the present study. Also, *S. cerevisiae* is one of the most widely used eukaryotic models to understand basic molecular processes in humans and other higher eukaryotes, it is an extraordinary workhorse for fermentation-based industrial applications, and is increasingly used for the toxicity assessment of substances, such as synthetic chemicals, heavy metals and engineered nanomaterials^24–30^. This study evaluates the toxicity of different GN concentrations for the yeast *S. cerevisiae*, through the analysis of cell viability, cytotoxicity, genotoxicity, and global transcriptional response.

## Results and Discussion

### Characteristics of the selected commercial polycarboxylate functionalized graphene nanoplatelets

The association between a certain biological response with the chemical and morphological properties of graphene requires an appropriate characterization of the product. The fate of graphene nanomaterials when exposed to biological systems is determined both by their intrinsic physicochemical characteristics such as lateral dimensions, thickness, and C/O ratio/functionalization and by their acquired characteristics upon contact with the biological environment, such as the biocorona^31^. In case of the selected commercial polycarboxylate functionalized graphene nanoplatelets (GN), in a recent study from our research group (Anton *et al*. 2018)^32^, it was determined that in contrast to graphene oxide, its ability to interact with biomolecules was very low. Also, the physical-chemical properties of GN were determined in the same study^32^. The product used by Anton *et al*. (2018) was exactly that used for this work (Sigma-Aldrich; ref: 806625; lot: MKBW5736V), therefore, the insights previously determined on its characteristics and properties are highly valuable for the present toxicology assessment. Microscopy analyses using AFM and TEM instruments showed that GN flakes were of variable size and appeared to be stacked in clusters from micrometric to nanometric scale. Also, diverse analytical techniques (ATR-FTIR, Raman, X-ray diffraction and XPS) were applied to understand the GN structure and composition. According to the XPS analysis, the material showed to have a very high carbon content (relative atomic composition close to 96%), with an oxygen composition of around 3.4%. In this regard, the presence of polycarboxylate groups, or any other oxygen containing functional groups could not be inferred from the ATR-FTIR spectra analysis, which suggests that their presence in the commercial product is neglectable. In fact, the dispersibility of the nanoparticles in water suspensions was low. Therefore, we did not consider the possible presence of carboxylic groups in the product during our toxicology assessment. Although the described analyses gave us good insights into the morphology and composition of this particular lot of commercial GN (ref: 806625; lot: MKBW5736V), none of these analytical techniques allowed the identification and quantification of trace elements^32^. However, since the presence of trace metal impurities in graphene derivatives, either contained in the graphite precursor or transferred by reactants used in the nanomaterial preparation, has been previously described^33–38^, a trace element analysis of GN was done by inductively coupled plasma mass spectrometry (ICP-MS) to fully characterize its composition. All metallic elements identified and their concentrations are displayed in Table 1.

**Table 1:** Composition of GN determined by ICP-MS.

	ppm
Al	2.17 ± 0.25
B	20.53 ± 7.20
Ba	9.03 ± 0.80
Ce	0.23 ± 0.02
Co	0.92 ± 0.07
Cr	62.85 ± 4.46
Cu	2.89 ± 0.60
Fe	276.03 ± 21.87
K	56800.80 ± 2143.97
Mg	11.95 ± 0.36
Mn	6.18 ± 0.37
Mo	6.17 ± 0.49
Na	730.53 ± 40.97
Nb	0.017 ± 0.002
Nd	0.016 ± 0.004
Ni	34.41 ± 2.54
Pr	0.007 ± 0.000
Pb	0.635 ± 0.043
Rb	8.23 ± 0.41
Si	323.63 ± 79.63
Sr	0.12 ± 0.03
V	0.084 ± 0.005
W	0.96 ± 0.38
Zr	0.335 ± 0.007

The reported values are the averages of two independent sample analyses.

A number of metallic elements, previously described as possible graphene contaminants, were identified^37–39^, such as silicon (323.63 ppm), iron (276.03 ppm), manganese (6.18 ppm), cobalt (0.92 ppm), copper (2.89 ppm), molybdenum (6.17 ppm) and nickel (34.41 ppm). The presence of K and Na is usual as well in graphene synthesis procedures based on chemical oxidation of graphite and subsequent thermal or chemical reduction^37,38^. The concentrations of most of the different elements identified in GN are relatively low, however, the presence of multi-metals should be carefully considered, due to the possible induction of mixture toxicity in biological systems directly exposed to the nanomaterial^40^.

### Determination of colony forming units *S. cerevisiae* cells exposed to different GN concentrations

The viability of *S. cerevisiae* cells exposed to two GN concentrations (160 and 800 mg L^−1^) and exposure times (2 and 24 h) was assessed through colony forming units (CFU) determination. The concentrations selected to assess the biological impact of GN on yeast cells were based on previous concentration ranges used by other authors in recent studies, where the impact of distinct graphene derivatives on fungal species was studied^41–44^. Also, a previous study provided toxicology data at transcriptomics level by exposing *S. cerevisiae* to 160 mg L^−1^ ^45^, so we considered interesting to use the mentioned concentration to assess and compare the toxicological response of the fungus to both nanoparticle types. Together with 160 mg L^−1^, we also decided to study the response of the fungus to a significantly higher concentration (5 times higher: 800 mg L^−1^), to compare the yeast response between two clearly contrasting conditions.

**Figure 1:**
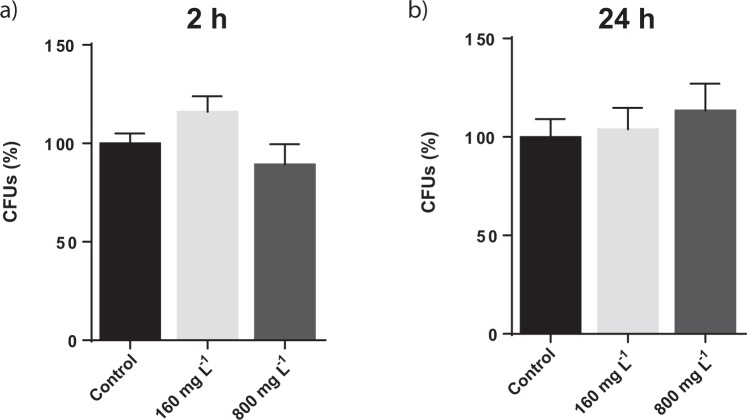
Colony forming units (CFUs) determination of *S. cerevisiae* cells exposed to 160 and 800 mg L^−1^ of GN during 2 hours (**a**) and 24 hours (**b**). The control condition corresponds to non-exposed *S. cerevisiae* cells. The reported values are the averages of three biological replicates per culture condition.

As displayed in Fig. 1, no differences in viability were observed in the selected exposure conditions. Therefore, the selected GN seem to have low toxicity towards *S. cerevisiae*, being at least lower than that reported for other carbon nanomaterials, such as 2D-graphene oxide (GO), 1D-multi-walled carbon nanotubes (MWCNTs) or 1D-oxidized single-walled carbon nanotubes (O-SWCNTs), which induced significant yeast growth inhibition at lower concentrations (160, 400 and 188.2 mg L^−1^ respectively)^41,44,46,47^. The impact of 0D-fullerene nanoparticles (nC60) exposure to *S. cerevisiae* was also studied, with no apparent effect on the growth yield of the fungus, although the nC60 concentration used (31 mg L^−1^) was lower than that used in the previously described studies^48^. The shape of carbon derived nanomaterials is a relevant characteristic influencing their interaction with biological molecules and organisms. However, other physicochemical features of carbon derived nanomaterials such as the chemical composition, size, stability, functionalization, charge, porosity and hydrophobicity/hydrophilicity, agglomeration or aggregation, also affect their reactivity^49^, making thus difficult to predict their toxicological potential in a particular organism by only considering their morphology.

The influence of graphene derivatives on the viability of other fungi has been little studied. Recently, two studies reported the impact of graphene oxide and reduced graphene oxide on the filamentous fungus *Phanerochaete chrysosporium*, with the latter compound showing lower toxicity for the fungus than the former^42,43^. Besides carbon nanomaterials, the toxicological impact on *S. cerevisiae* of various metal oxide nanoparticles have been evaluated as well, generally showing low toxicity^50–52^.

### Determination of oxidative stress

**Figure 2:**
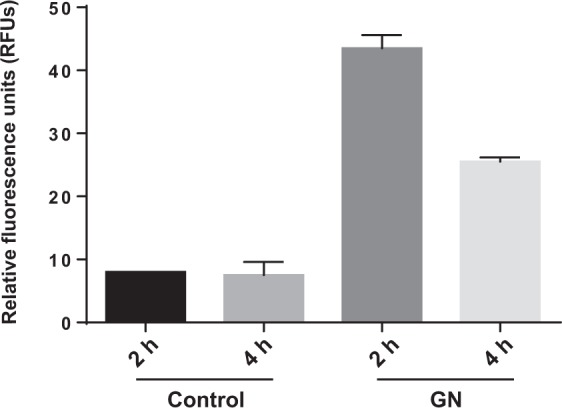
Oxidative stress (ROS) determination of *S. cerevisiae* cells exposed to 160 mg L^−1^ of GN during 2 and 4 hours. The control condition corresponds to non-exposed *S. cerevisiae* cells. The reported values are the averages of two biological replicates per culture condition.

To evaluate whether GN were able to induce oxidative stress in *S. cerevisiae*, cells growing at exponential phase were exposed to 160 mg L^−1^ of the nanomaterial, for 2 and 4 hours. As shown in Fig. 2, the oxidative stress levels were significantly increased in *S. cerevisiae* in the presence of the carbon derived nanoparticle. Reactive oxygen species (ROS) levels were significantly higher 2 hours after the exposure started, but also remained significantly higher than in the negative control at 4 hours. Carbon derived nanomaterials have shown previously to induce oxidative stress in yeast. In case of GO and O-SWCNT, similar concentrations to the one tested here also induced ROS, but the exposure time tested in both cases was 24 hours, while no clear evidence of ROS formation was observed for yeast cells exposed to MWCNT^41,46,47^. In a more recent study, the induction of oxidative stress in yeast at short exposure periods (2 and 4 hours) by different commercial GO products was also determined^44^.

### Determination of genotoxic effect

**Figure 3:**
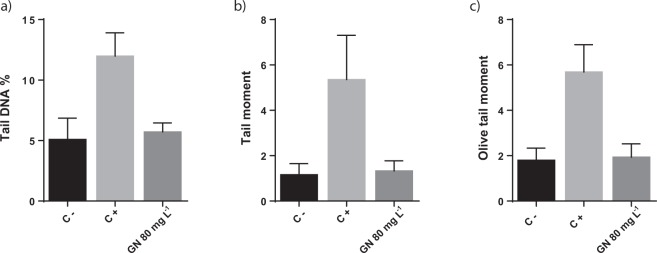
Comet assay for DNA damage analysis on *S. cerevisiae* cells through the quantification of tail DNA % (**a**), tail moment (**b**) and olive tail moment (**c**) parameters in non-exposed cells (negative control; C−), cells exposed to 10 mM of H_2_O_2_ (positive control; C+) and cells exposed to 80 mg L^−1^ of GN. The reported values are the averages of three biological replicates per culture condition.

The possible genotoxic effect of the selected GN on *S. cerevisiae* cells was determined following a standard protocol previously described^53^. GN concentrations higher than 80 mg L^−1^ could not be tested using the described methodology, as the cell nuclei could not be properly visualized under the fluorescence microscope. Therefore, the potential genotoxic effect of GN on *S. cerevisiae* spheroplasts was determined at 80 mg L^−1^. At least 450 comets per condition were analysed, and the parameters tail DNA, tail moment and olive tail moment, previously applied in similar studies done in *S. cerevisiae*^54,55^, were calculated to estimate the genotoxic potential of GN. As it can be observed in Fig. 3, no significant differences in any of the three parameters were observed between the negative control and the conditions were *S. cerevisiae* was exposed to the graphene nanoparticles.

Results obtained in the colony forming units, oxidative stress and genotoxicity analyses indicate that, while high concentrations of GN (up to 800 mg L^−1^) at long exposure periods of time (up to 24 hours) were not able to reduce *S. cerevisiae* cells viability, the nanoparticles could provoke oxidative stress at early culture stages (2 hours) and at a lower concentration (at least 160 mg L^−1^), while no signs of induced genotoxicity were observed in the selected conditions. The observation of ROS production without an apparent impact on cell viability is not rare, however both oxidative stress and viability loss are usually observed for graphene derivatives in similar exposure experiments^41,44,56–58^.

### Transcriptional response of *S. cerevisiae* cells to different GN concentrations

Having into consideration the above presented results, a transcriptomics experiment was performed to assess the early response (2 hours) of *S. cerevisiae* cells exposed to different GN concentrations (160 and 800 mg L^−1^). The aim of this experiment was to understand the early response of yeast to this type of carbon nanoparticle, for which no similar studies have been reported so far. Additionally, we also wanted to pinpoint the molecular mechanisms behind the early oxidative damage induced by GN to identify possible toxicity pathways.

Differently to what was observed in previous studies for GO^41^, the ability of GN to bind RNA was very low, so it was possible to isolate total RNA from *S. cerevisiae* cells exposed to this type of nanomaterial without having to introduce a particle-cells separation process. After RNA isolation, the integrity of the purified ribonucleic acids was analyzed through an agarose gel based visualization analysis, and by analyzing the samples with a bioanalyzer (Agilent 2100). RNA-Seq analysis was done using the Illumina sequencing system (further details can be found in the Materials and Methods section). Once the RNA-Seq reads were obtained and mapped to the *S. cerevisiae* BY4741 strain genome, information regarding the mapping status could be visualized (Supplementary Table S1). In all cases, the amount of total reads that mapped the *S. cerevisiae* genome ranged between 91.3 and 93.5%. This result, together with the total reads obtained for each of the samples and the fact that around 87.7% to 92.7% of the reads mapped to exonic regions in the genome, gave a good indication about the high quality of the RNA generated in this experiment.

**Figure 4:**
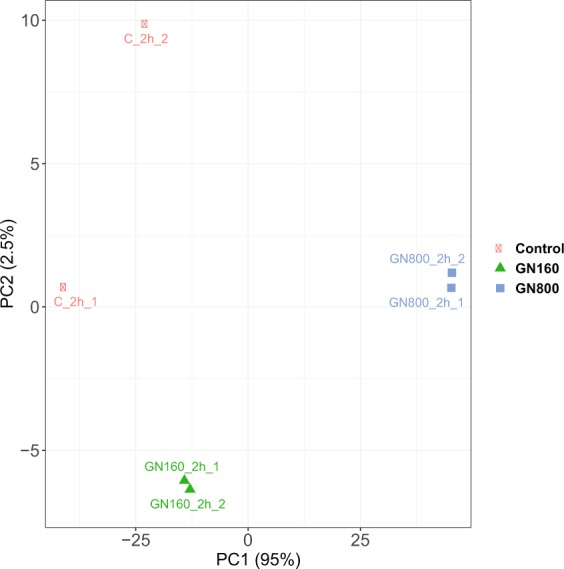
Principal Component Analysis (PCA) plot of the biological replicates of transcripts from three different exposure conditions to polycarboxylate functionalized graphene nanoplatelets (non-exposed, 160 mg L^−1^ and 800 mg L^−1^).

After read mapping and normalization, Principal Component Analysis (PCA) was used to represent the variability between samples and among biological replicates. The results show that the samples clustered together in a condition specific manner, with only minor variations between the independent biological replicates (Fig. 4). The GN800 condition displayed higher levels of transcriptomic change condition compared to GN160 in relation to the control condition, as evident in the separation of the samples along PC1, which explains a large part (95%) of the variance in the data.

**Figure 5:**
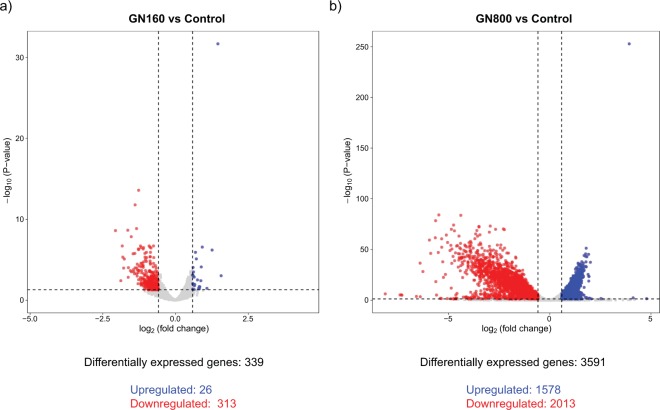
Volcano plots displaying the amount of differentially expressed genes and fold change (log_2_) in expression levels in GN160 (a) and GN800 (b) conditions when compared to the control condition. Genes were considered differentially expressed if FC > 1.5 (upregulated) or FC < 1/1.5 (downregulated) and false discovery rate (FDR) was lower than 0.05.

Regarding the differential expression of genes between GN160 vs C and GN800 vs C (Supplementary Table S2), volcano plots were obtained (Fig. 5). We only consider as differentially expressed with a biological meaning those genes with a difference in expression higher than 1.5-fold (corresponding to 0.585 log_2_ FC) and p-value (after correction for multiple testing) lower than 0.05. As it can be observed in Fig. 5, the number of differentially expressed genes in *S. cerevisiae* was widely different. In the presence of 160 mg L^−1^ of GN, 339 genes were differentially expressed between the exposed and non-exposed cells, while cells exposed to a GN concentration 5 times higher (800 mg L^−1^) had 3591 differentially expressed genes. Therefore, the number of genes showing altered expression specifically in the presence of 800 mg L^−1^ of GN was much higher, showing that the presence of a higher concentration of the nanomaterial induces a stronger transcriptional response in *S. cerevisiae*. In both GN-exposed conditions, most of the genes showing significant changes in their expression levels were downregulated. This was particularly remarkable in case of the GN160 condition, where 313 out of the observed 339 differentially expressed genes were downregulated. Both GN concentrations induced expression changes in a common group of genes (337), while a high number of genes were only differentially expressed in the GN800 condition (3254). It is interesting to remark that virtually all genes that showed a significant expression change in GN160, were also differentially regulated in GN800 as well (337 out of 339 genes).

In relation to the biological processes which show an altered functionality in the conditions where *S. cerevisiae* was exposed to different GN concentrations, a Gene Ontology^59,60^ enrichment and a KEGG (Kyoto Encyclopedia of Genes and Genomes)^61^ pathway enrichment analysis of differentially expressed genes was done. Results of the enrichments in categories related to biological process (BP), molecular function (MF) and cellular component (CC) are provided in the supplementary material (Supplementary Table S3).

Metabolic pathway enrichment also highlights the upregulation of genes associated to core metabolic processes such as synthesis and utilisation of sugars, amino acids, lipids, organic acids, hormones, etc. Table 2 summarizes the significant pathways enriched in the up and down regulation of genes upon both exposures (full results can be found in Supplementary Table S4).

**Table 2:** Metabolic pathway enrichment analysis.

	Size	Commonly upregulated genes (GN160 and GN800) vs Control (26 genes)			GN800 upregulated genes vs Control (1578 genes)		
		# in sample	p-value	FDR	# in sample	p-value	FDR
Glycolysis/Gluconeogenesis	56	3	0.001	0.008	33	2.58E-09	4.13E-08
Citrate cycle (TCA cycle)	32	0	NA	NA	21	1.58E-07	2.21E-06
Pentose phosphate pathway	28	0	NA	NA	15	0.000	0.002
Fructose and mannose metabolism	23	2	0.003	0.015	10	0.018	0.057
Galactose metabolism	25	2	0.004	0.015	9	0.081	0.161
Steroid biosynthesis	17	0	NA	NA	9	0.005	0.023
Alanine, aspartate and glutamate metabolism	32	0	NA	NA	16	0.000	0.003
Glycine, serine and threonine metabolism	31	0	NA	NA	15	0.001	0.007
Cysteine and methionine metabolism	40	0	NA	NA	20	9.58E-05	0.001
Valine, leucine and isoleucine degradation	13	0	NA	NA	10	4.06E-05	0.000
Valine, leucine and isoleucine biosynthesis	12	1	0.043	0.072	9	0.000	0.001
Lysine biosynthesis	12	0	NA	NA	8	0.001	0.007
Arginine and proline metabolism	21	0	NA	NA	10	0.009	0.036
Phenylalanine, tyrosine and tryptophan biosynthesis	17	0	NA	NA	11	0.000	0.001
beta-Alanine metabolism	13	0	NA	NA	7	0.012	0.042
Glutathione metabolism	25	0	NA	NA	11	0.012	0.042
Starch and sucrose metabolism	41	2	0.010	0.022	22	1.01E-05	0.000
Other types of O-glycan biosynthesis	13	0	NA	NA	7	0.012	0.042
Amino sugar and nucleotide sugar metabolism	33	2	0.006	0.020	15	0.002	0.012
Glycerophospholipid metabolism	38	0	NA	NA	15	0.012	0.042
alpha-Linolenic acid metabolism	3	0	NA	NA	3	0.011	0.042
Pyruvate metabolism	43	3	0.000	0.008	24	1.55E-06	1.74E-05
Propanoate metabolism	13	1	0.046	0.072	7	0.012	0.042
Butanoate metabolism	9	0	NA	NA	6	0.005	0.023
Methane metabolism	26	0	NA	NA	13	0.002	0.009
2-Oxocarboxylic acid metabolism	35	1	0.120	0.138	21	1.41E-06	1.74E-05
Biosynthesis of amino acids	121	2	0.071	0.095	72	7.77E-16	2.18E-14
MAPK signaling pathway – yeast	114	1	0.343	0.343	55	5.37E-10	1.00E-08
Mitophagy - yeast	41	0	NA	NA	17	0.004	0.020
Hippo signaling pathway - multiple species	8	0	NA	NA	6	0.002	0.011
		**Commonly downregulated genes (GN160 and GN800) vs Control (311 genes)**			**GN800 downregulated genes vs Control (2013 genes)**		
Oxidative phosphorylation	72	7	0.0379	0.2877	33	0.0011	0.0179
Ribosome	183	19	0.0004	0.0076	116	2.22E-16	2.26E-14
RNA polymerase	30	6	0.0017	0.0211	14	0.0241	0.2727
Spliceosome	79	12	0.0002	0.0057	36	0.0007	0.0179
Proteasome	35	1	0.7932	0.9699	19	0.0010	0.0179
Protein export	22	2	0.2515	0.7326	15	0.0001	0.0058
Endocytosis	74	6	0.1055	0.5011	34	0.0008	0.0179

Size indicates number of genes in the genome assigned to the pathway, whereas #in sample refer to the considered set. Only pathways with FDR < 0.05 in some of the sets have been kept. NA indicates not available.

Amongst the common significantly upregulated genes in the two exposure conditions (26) some associated to increased sugar metabolism were found, although the relatively low number of genes associated to this biological process precluded additional analysis. Four genes were associated to the term GO 0042221 (“response to toxin”): YNL134C, YDR533C (*HSP31*), YKR076W (*ECM4*) and YOL151W (*GRE2*). The gene with accession number YNL134C is a NADH-dependent aldehyde reductase, involved in detoxification of furfural, with a broad substrate specificity^62^; Hsp31p is a heat-shock stress response protein, which confers protection against reactive oxygen species^63^; *ECM4* codes for a cell wall glutathione transferase whose expression has been shown to be upregulated upon exposure to genotoxic agents, such as methyl methanesulfonate, cisplatin and bleomycin^64^; and *GRE2* codes for a 3-methylbutanal reductase and NADPH-dependent methylglyoxal reductase, whose expression is positively affected by oxidative stress, and it is regulated by the HOG pathway, a mitogen-activated protein kinase (MAPK) pathway mainly related to hyperosmotic stress response in *S. cerevisiae*^65^. Additional genes having a function related to oxidation-reduction processes (YCR102C, YLR460C, YML131W, YJL052W *(TDH1)*) were upregulated too in GN160 and GN800 compared to the control: YCR102C, YLR460C and YML131W belong to the medium-chain dehydrogenase/reductase (MDR) family, which includes metabolic enzymes acting on alcohols or aldehydes, with possible roles in detoxifying alcohols and related compounds, protecting against environmental stresses such as osmotic shock, reduced or elevated temperatures, or oxidative stress^66^, while *TDH*1 codes for a glyceraldehyde-3-phosphate dehydrogenase (GAPDH) isozyme, whose expression is regulated by reductive stress caused by an excess of cytoplasmic NADH^67^. Detailed inspection of the results also showed upregulation of YDL085W (*NDE2*) in the presence of both GN concentrations, although the change has a FDR slightly above the selected threshold (FDR = 0.057). This gene codes for a mitochondrial external NADH dehydrogenase involved in providing the cytosolic NADH to the mitochondrial respiratory chain^68^. The multidrug efflux pump coding gene YML116W (*ATR1*), which confers resistance to aminotriazole, 4-nitroquinoline-N-oxide and 5-fluorouracil^69,70^, and whose expression has been found to increase during DNA-replication stress^70^, was also found to be upregulated in both GN160 and GN800. This was also the case for the endochitinase coding gene YLR286C (*CTS1*), involved in cell separation^71^, and the genes YCL040W (*GLK1*) and YFR053C (*HXK1*), related to carbohydrate metabolic process. The above described genes, upregulated in the presence of lower (160 mg L^−1^) and higher (800 mg L^−1^) GN concentrations, are the core response activated by *S. cerevisiae*, whose overexpression can be associated to a fungal response to reduce the nanoparticles toxicity and to maintain cell homeostasis.

Regarding the common significantly downregulated genes in both exposure conditions, most of them are related to cell cycle, protein complex biogenesis, ribosome biogenesis and RNA processing and metabolism. Previous toxicology studies in *S. cerevisiae* have observed a significant downregulation of ribosomal biogenesis and assembly genes in response to different stresses^72–74^ and the same has been reported in case of RNA processing genes^75^.

As mentioned earlier, exposing *S. cerevisiae* to the higher GN concentration induced a very strong transcriptional response of the fungus. The number of specifically upregulated genes in the presence of 800 mg L^−1^ of GN was very high (1578), and this produced changes in many different gene networks. Many of the transcriptional changes occurred in metabolic genes, related to the synthesis and utilisation of sugars, amino acids, lipids and key metabolic process related to energy and redox balances such as the TCA cycle or the pentose phosphate pathway (see Table 2). A group of upregulated genes showed the same behaviour when *S. cerevisiae* cells were exposed to other nanocarbon derivatives, suggesting the existence of a common biological response to different nanomaterials. For instance, the superoxide dismutase genes *SOD1* (YJR104C) and *SOD2* (YHR008C), which play a role in oxygen radical detoxification, and *YCA1* (YOR197W), involved in apoptosis regulation, were found to be upregulated too when yeast cells were exposed to different MWCNTs concentrations^46^. Additionally, several iron transport and metabolism related genes (YHL040C (*ARN1*), YOR382W (*FIT2*), YOR383C (*FIT3*), YER145C (*FTR1*), YMR058W (FET3) and YOR384W (*FRE5*)) were also overexpressed in the presence of graphene oxide^45^. GO was reported to induce the disruption of yeast iron-related physiological and metabolic processes when present in the environment at 160 mg L^−1^ ^45^, but we did not observe the same biological response when using the same concentration of GN. However, based on our observations, GN could cause extracellular iron deficiency as well in yeast when present in higher concentrations (800 mg L^−1^). In fact, besides the mentioned iron utilisation genes, a high number of additional ORFs related to metal ion transport and homeostasis showed significantly higher expression levels in GN800, indicating that high concentrations of the nanomaterial reduce the bioavailability of metallic elements for the fungus. Considering these observations and the metals and metalloids concentration determined in GN by ICP-MS, the possibility that the selected nanomaterials exert metal induced toxicity on *S. cerevisiae* is low.

The pathway enrichment analysis does not allow to distinguish between amino acid biosynthesis and utilization, as many enzymes are involved in both processes. However detailed inspection of the genes suggests increased degradation in the GN800 condition. For instance, the arginine related regulator YMR042W (*ARG80*) and its targets, the arginine catabolic genes YPL111W (*CAR1*) and YLR438W (*CAR2*)^76^, appear upregulated in the presence of 800 mg L^−1^ of GN. The increased amino acid degradation is consistent with the reduced expression of genes associated to translation and protein synthesis.

We also observed an activation of the glutamate dehydrogenase (GDH) pathway, indicated by the upregulation of the ammonia permease coding gene YGR121C (*MEP1*), YOR375C (*GDH1*) and YOR375C (*GDH3*), coding for two NADP-dependent GDH isoforms for glutamate synthesis, and the glutamine synthase (GS) gene YPR035W (*GLN1*), and for the reduced expression of YDL215C (*GDH2*), another NADP-dependent GDH responsible for glutamate degradation. The GDH pathway is known to be regulated by the quality and availability of nitrogen and carbon sources^77^. Upon exposure to 800 mg L^−1^ of GN, two of the main regulators of the nitrogen catabolite repression (NCR) pathway, YFL021W (*GAT1*) and YER040W (*GLN3*) appear overexpressed as well as two of the main regulators associated to sugar catabolite repression (YGL035C) *MIG1* and (YGL035C) *GAL80*. The GDH pathway is also related to response to stress, as GDH3 is needed for resistance to ROS stress induced apoptosis^78^. The GDH pathway leads to glutamate synthesis which is the starting point for gamma-glutamylcysteine and glutathione synthesis. The analysis shows a significant enrichment in genes in the glutathione synthesis pathway. Gamma-glutamylcysteine and glutathione are potent antioxidants^79^, so the upregulation of this pathway further indicates a general response to counteract possible oxidative stress induced by the high GN concentration.

Also, changes related to cell reproduction, filamentous growth and cell aggregation were observed. These changes indicate that high GN concentrations induce severe changes in the physiological state of the yeast. The fact that many upregulated genes are related to “aging” (29), could indicate that higher concentrations of GN induce early senescence or cell death. Also, fact that many genes related to osmotic stress (35) and membrane invagination (57), indicate that the presence of high GN concentrations suppose an environmental thread for the stability and integrity of the fungus.

## Conclusion

The toxicity assessment of commercial polycarboxylate functionalized graphene nanoplatelets using the model fungus *S. cerevisiae* has unveiled the potential impact of this type of nanomaterial to rapidly alter the physiological state of the yeast. Overall, GN showed to have low lethal toxicity levels for *S. cerevisiae*, although it was capable to induce oxidative stress at the lower concentration tested, indicating the potential of the nanomaterial to provoke cellular damage. The analysis of the transcriptional landscape of *S. cerevisiae* cells exposed to different GN concentrations indicated that the yeast was forced to induce detoxification and oxidative stress responses in the presence of the nanomaterial, and severe changes in its physiological state were observed too. The reported results contribute to the understanding of the molecular mechanisms underlying yeast-graphene interactions, which could influence the performance of applications based on interfacing cells with the nanomaterial, and give an indication of the exposure risk of unicellular eukaryotic organisms. Also, the reported results highlight the complexity of microbial systems-graphene interactions.

## Methods

### Materials, reagents and strains

Most of the chemicals and reagents were purchased to Sigma-Aldrich and Acros Organics. In particular, the polycarboxylate functionalized graphene nanoplatelets (ref: 806625; lot: MKBW5736V) were purchased to Sigma-Aldrich. The *S. cerevisiae* BY4741 strain was purchased to Thermo Fisher. Yeast cells were grown and maintained in standard liquid YPD medium (1% yeast extract, 1% yeast bacto-peptone, 2% glucose). Cell cultures in liquid media were kept on a rotary shaker at 185 rpm at 30 °C.

### ICP-MS

Metals and metalloids content in GN were determined following the protocol reported by Domi *et al*. (2020) with minor changes^44^. Graphene samples (0.1 g) were subjected to a digestion process with 7 mL of HNO_3_ Suprapur (Merck) (65% v/v) and 1 ml of H_2_O_2_ (30% v/v), while being subjected to the following thermal treatment: a temperature gradient from room temperature up to 80 °C in 4 minutes, followed by a second temperature gradient, from 80 °C to 120 °C in 4 minutes, and by a third temperature gradient, from 120 °C to 190 °C in 5 minutes. Then, temperature was kept constant at 190 °C for 30 minutes, and finally samples were cooled down for 1 hour. The analysis of digested samples was done with an Agilent 8900 ICP-QQQ instrument.

### Determination of oxidative stress

Intracellular levels of reactive oxygen species (ROS) were determined using the reagent CM-H2DCFDA, following a protocol similar to that reported by James *et al*. (2015)^80^. *S. cerevisiae* cells growing in exponential phase were pelleted, washed and incubated with CM-H2DCFDA (7 μM) in PBS for 60 minutes, at 30 °C and 185 rpm. Afterwards, yeast cells were washed again, resuspended in YPD and subsequently exposed to the GN nanomaterial (160 mg L^−1^) for 2 and 4 hours. Then, yeast cells were washed two times with PBS, incubated 2 minutes in a solution containing AcLi 2 M, and subsequently washed and incubated again for 2 minutes in a solution containing SDS (0.01%) and chloroform (0.4%). Finally, cells were pelleted and the supernatant was transferred to a black opaque 96 micro-well plate, where fluorescence was measured using a microplate reader (BioTek Synergy HT, excitation wavelength, 485 nm; emission wavelength 528 nm).

### Yeast comet assay

The yeast comet assay was done following the protocol published by Oliveira and Johansson (2012)^81^. Yeast spheroplasts were exposed to 80 mg L^−1^ of GN during 40 minutes at 4 °C. Three biological replicates were analysed per culture condition. A Leica DMI6000 B inverted fluorescence microscope was used to analyse the obtained microgels, to visualize and register the yeast comets. At least 150 comets were registered per biological replicate and subsequently analysed with the open access software CASP^82^.

### RNA isolation, quality control and sequencing

RNA isolation was performed using Thermo Fisher Scientific reagents, following the TRIzol Plus RNA Purification Kit user guide (Pub. No. MAN0000561), with minor modifications^41,83^. Briefly, yeast aliquots were pelleted by centrifugation (13000 g) and subsequently resuspended in 1 mL of TRIzol reagent in a 2 mL tube, prefilled with glass beads (MP). Yeast samples were disrupted using a FastPrep-24 Instrument (MP). After disruption, 200 µL of chloroform were added and the mix was homogenated for 10 seconds. The mix was poured into Phasemaker tubes (2 mL), and centrifuged at 13000 g in a table-top centrifuge^83^. The RNA present in the water phase was purified using the PureLink RNA Mini Kit (Thermo), following the manufacturer’s instructions. RNA integrity was assessed with an Agilent 2100 system, and only high quality samples (RIN value ≥8) were selected for whole transcriptome shotgun sequencing^83^. Total RNA was sent for whole transcriptome sequencing to Novogene Bioinformatics Technology Co. Ltd. (HongKong, China). mRNA sequencing (RNA-Seq) was performed using Ilumina Hiseq. 4000 and the Casava pipeline version 1.8.2.

### RNA-Seq data processing and analysis

Reads were pre-processed using FastqPuri for quality control and adapter, contamination and quality filtering^84^. Reads with adapter contamination were removed, as well as the ones with 50% of the bases with quality below 20. Also, reads with a percentage of unidentified bases greater than 10% were also removed. Latest assembly of the reference genome for this strain was retrieved from Ensembl^85^, genome accession number (GCA_000146045.2). Reads were mapped to the genome using Star v2.7^86^. The genome was indexed specifying the read length to improve accuracy. The mapping was done using two pass method. Number of reads for each genome feature were retrieved using featureCounts^87^. Total number of reads are summarized in the supplementary material (Supplementary Table S1). Data have been submitted to the European Nucleotide Archive and can be found under the accession number PRJEB33532.

Read counts per gene were normalized and differential expression was computed using DESeq. 2 v 1.24^88^, with default parameters except for the alpha threshold that was set to 0.05. Variance stabilizing transformation considering the experimental design was performed using the ‘vst’ command prior to principal component analysis (PCA). Enrichment analysis for selected groups of genes were performed using the hypergeometric function to model the background probability and the Benjamini–Hochberg procedure was used to control the false discovery rate (FDR) and correct for multiple testing. Gene ontology (GO) enrichment analysis was performed using the BINGO Cytoscape app (v 3.0.3)^89^. Annotation to be used with BINGO was downloaded from the Gene Ontology^59,60^. For the metabolic pathway enrichment analysis, gene to pathway associations were retrieved from KEGG^61,90^, and all genes in the genome were used as a background set. Statistical manipulations and graphical representations of the data were performed using R (v 3.6.1)^91^, and the packages ggplot2 (v3.2.0) ^92^. Further information related to the identified differentially expressed genes was obtained using The Saccharomyces Genome Database (SGD)^93^.

## Supplementary Information

Supplementary Information.

Supplementary Table S1.

Supplementary Table S2.

Supplementary Table S3.

Supplementary Table S4.

## References

[1] Bianco A (2013). Graphene: Safe or toxic? the two faces of the medal. Angewandte Chemie - International Edition.

[2] Kauling AP (2018). The Worldwide Graphene Flake Production. Advanced Materials.

[3] Tadyszak, K., Wychowaniec, J. K. & Litowczenko, J. Biomedical Applications of Graphene-Based Structures. *Nanomaterials (Basel, Switzerland)* **8**, (2018). 10.3390/nano8110944 PMC6267346 30453490

[4] Singh DP (2018). Graphene oxide: An efficient material and recent approach for biotechnological and biomedical applications. Materials Science and Engineering: C.

[5] Arvidsson R, Boholm M, Johansson M, de Montoya ML (2018). “Just Carbon”: Ideas About Graphene Risks by Graphene Researchers and Innovation Advisors. Nanoethics.

[6] Ou L (2016). Toxicity of graphene-family nanoparticles: a general review of the origins and mechanisms. Particle and fibre toxicology.

[7] Ema M, Gamo M, Honda K (2017). A review of toxicity studies on graphene-based nanomaterials in laboratory animals. Regulatory Toxicology and Pharmacology.

[8] Sanchez VC, Jachak A, Hurt RH, Kane AB (2012). Biological Interactions of Graphene-Family Nanomaterials: An Interdisciplinary Review. Chemical Research in Toxicology.

[9] Karahan HE (2018). Antimicrobial graphene materials: the interplay of complex materials characteristics and competing mechanisms. Biomaterials Science.

[10] Zarafu I (2018). Antimicrobial Features of Organic Functionalized Graphene-Oxide with Selected Amines. Materials.

[11] Zhao R (2018). Highly Stable Graphene-Based Nanocomposite (GO–PEI–Ag) with Broad-Spectrum, Long-Term Antimicrobial Activity and Antibiofilm Effects. ACS Applied Materials & Interfaces.

[12] Jaworski S (2018). Graphene Oxide-Based Nanocomposites Decorated with Silver Nanoparticles as an Antibacterial Agent. Nanoscale Research Letters.

[13] Li N (2018). Powerful antibacterial activity of graphene/nanoflower-like nickelous hydroxide nanocomposites. Nanomedicine.

[14] Li C (2013). The antifungal activity of graphene oxide–silver nanocomposites. Biomaterials.

[15] Farzanegan A (2018). Synthesis, characterization and antifungal activity of a novel formulated nanocomposite containing Indolicidin and Graphene oxide against disseminated candidiasis. Journal de Mycologie Médicale.

[16] Ficociello Graziella, De Caris Maria, Trillò Giusy, Cavallini Domenico, Sarto Maria, Uccelletti Daniela, Mancini Patrizia (2018). Anti-Candidal Activity and In Vitro Cytotoxicity Assessment of Graphene Nanoplatelets Decorated with Zinc Oxide Nanorods. Nanomaterials.

[17] Asadi Shahi S, Roudbar Mohammadi S, Roudbary M, Delavari H (2019). A new formulation of graphene oxide/fluconazole compound as a promising agent against Candida albicans. Progress in biomaterials.

[18] Yang SH (2012). Interfacing Living Yeast Cells with Graphene Oxide Nanosheaths. Macromolecular Bioscience.

[19] Khanra P (2012). Simultaneous bio-functionalization and reduction of graphene oxide by baker’s yeast. Chemical Engineering Journal.

[20] Valentini L, Bittolo Bon S, Signetti S, Pugno NM (2016). Graphene-Based Bionic Composites with Multifunctional and Repairing Properties. ACS Applied Materials & Interfaces.

[21] Kempaiah R, Chung A, Maheshwari V (2011). Graphene as Cellular Interface: Electromechanical Coupling with Cells. ACS Nano.

[22] Kubacka A (2015). Understanding the antimicrobial mechanism of TiO2-based nanocomposite films in a pathogenic bacterium. Scientific Reports.

[23] Wu J (2012). Polycarboxylation of carbon nanofibers under Friedel–Crafts condition: A simple route to direct binding of carboxylic functionalities to graphitic π-system. Chemical Physics Letters.

[24] Bao S, Lu Q, Fang T, Dai H, Zhang C (2015). Assessment of the toxicity of CuO nanoparticles by using Saccharomyces cerevisiae mutants with multiple genes deleted. Applied and Environmental Microbiology.

[25] Mager WH, Winderickx J (2005). Yeast as a model for medical and medicinal research. Trends in Pharmacological Sciences.

[26] Duina AA, Miller ME, Keeney JB (2014). Budding Yeast for Budding Geneticists: A Primer on the *Saccharomyces cerevisiae* Model System. Genetics.

[27] Mattanovich D, Sauer M, Gasser B (2014). Yeast biotechnology: teaching the old dog new tricks. Microbial Cell Factories.

[28] Nomura T (2013). Exposure of the Yeast *Saccharomyces cerevisiae* to Functionalized Polystyrene Latex Nanoparticles: Influence of Surface Charge on Toxicity. Environmental Science & Technology.

[29] Kitagawa Emiko, Momose Yuko, Iwahashi Hitoshi (2003). Correlation of the Structures of Agricultural Fungicides to Gene Expression inSaccharomyces cerevisiaeupon Exposure to Toxic Doses. Environmental Science & Technology.

[30] Kasemets K, Käosaar S, Vija H, Fascio U, Mantecca P (2019). Toxicity of differently sized and charged silver nanoparticles to yeast *Saccharomyces cerevisiae* BY4741: a nano-biointeraction perspective. Nanotoxicology.

[31] Fadeel B (2018). Safety Assessment of Graphene-Based Materials: Focus on Human Health and the Environment. ACS Nano.

[32] Antón-Millán N (2018). Influence of Three Commercial Graphene Derivatives on the Catalytic Properties of a *Lactobacillus plantarum* α- l -Rhamnosidase When Used as Immobilization Matrices. ACS Applied Materials & Interfaces.

[33] Ambrosi A (2012). Chemically reduced graphene contains inherent metallic impurities present in parent natural and synthetic graphite. Proceedings of the National Academy of Sciences of the United States of America.

[34] Lupina G (2015). Residual Metallic Contamination of Transferred Chemical Vapor Deposited Graphene. ACS Nano.

[35] Lisi N (2017). Contamination-free graphene by chemical vapor deposition in quartz furnaces. Scientific Reports.

[36] Ye R (2018). Manganese deception on graphene and implications in catalysis. Carbon.

[37] Wong CHA (2014). Synthetic routes contaminate graphene materials with a whole spectrum of unanticipated metallic elements. Proceedings of the National Academy of Sciences of the United States of America.

[38] Mazánek, V. *et al*. Ultrapure Graphene Is a Poor Electrocatalyst: Definitive Proof of the Key Role of Metallic Impurities in Graphene-Based Electrocatalysis. *ACS Nano*, 10.1021/acsnano.8b07534 (2019). 10.1021/acsnano.8b07534 30624902

[39] Jalili R (2018). Silicon as a ubiquitous contaminant in graphene derivatives with significant impact on device performance. Nature Communications.

[40] Mesquita VA, Silva CF, Soares EV (2016). Toxicity Induced by a Metal Mixture (Cd, Pb and Zn) in the Yeast Pichia kudriavzevii: The Role of Oxidative Stress. Current Microbiology.

[41] Zhu S, Luo F, Zhu B, Wang G-X (2017). Toxicological effects of graphene oxide on Saccharomyces cerevisiae. Toxicology research.

[42] Yang H (2018). Influence of reduced graphene oxide on the growth, structure and decomposition activity of white-rot fungus *Phanerochaete chrysosporium*. RSC Advances.

[43] Xie J (2016). Toxicity of graphene oxide to white rot fungus Phanerochaete chrysosporium. Chemosphere.

[44] Domi B (2019). Interaction Analysis of Commercial Graphene Oxide Nanoparticles with Unicellular Systems and Biomolecules. International Journal of Molecular Sciences.

[45] Yu Q (2017). Graphene oxide significantly inhibits cell growth at sublethal concentrations by causing extracellular iron deficiency. Nanotoxicology.

[46] Zhu S (2016). Toxicological effects of multi-walled carbon nanotubes on Saccharomyces cerevisiae: The uptake kinetics and mechanisms and the toxic responses. Journal of Hazardous Materials.

[47] Zhu S, Luo F, Li J, Zhu B, Wang G-X (2018). Biocompatibility assessment of single-walled carbon nanotubes using Saccharomyces cerevisiae as a model organism. Journal of Nanobiotechnology.

[48] Hadduck AN, Hindagolla V, Contreras AE, Li Q, Bakalinsky AT (2010). Does aqueous fullerene inhibit the growth of saccharomyces cerevisiae or escherichia coli?. Applied and Environmental Microbiology.

[49] Madannejad R (2019). Toxicity of carbon-based nanomaterials: Reviewing recent reports in medical and biological systems. Chemico-Biological Interactions.

[50] Kasemets K, Ivask A, Dubourguier H-C, Kahru A (2009). Toxicity of nanoparticles of ZnO, CuO and TiO2 to yeast Saccharomyces cerevisiae. Toxicology in Vitro.

[51] Kasemets K, Suppi S, Künnis-Beres K, Kahru A (2013). Toxicity of CuO Nanoparticles to Yeast *Saccharomyces cerevisiae* BY4741 Wild-Type and Its Nine Isogenic Single-Gene Deletion Mutants. Chemical Research in Toxicology.

[52] García-Saucedo C, Field JA, Otero-Gonzalez L, Sierra-Álvarez R (2011). Low toxicity of HfO2, SiO2, Al2O3 and CeO2 nanoparticles to the yeast, Saccharomyces cerevisiae. Journal of Hazardous Materials.

[53] Oliveira R, Johansson B (2012). Quantitative DNA damage and repair measurement with the yeast comet assay. Methods in Molecular Biology.

[54] Rank J, Syberg K, Jensen K (2009). Comet assay on tetraploid yeast cells. Mutation Research/Genetic Toxicology and Environmental Mutagenesis.

[55] Bayat N, Rajapakse K, Marinsek-Logar R, Drobne D, Cristobal S (2014). The effects of engineered nanoparticles on the cellular structure and growth of Saccharomyces cerevisiae. Nanotoxicology.

[56] Ou L (2017). The mechanisms of graphene-based materials-induced programmed cell death: a review of apoptosis, autophagy, and programmed necrosis. International journal of nanomedicine.

[57] Chang Y (2011). *In vitro* toxicity evaluation of graphene oxide on A549 cells. Toxicology Letters.

[58] Mittal S (2016). Physico-chemical properties based differential toxicity of graphene oxide/reduced graphene oxide in human lung cells mediated through oxidative stress. Scientific Reports.

[59] Ashburner M (2000). Gene Ontology: tool for the unification of biology. Nature Genetics.

[60] The Gene Ontology Consortium. (2019). The Gene Ontology Resource: 20 years and still GOing strong. Nucleic Acids Research.

[61] Kanehisa M (2000). KEGG: Kyoto Encyclopedia of Genes and Genomes. Nucleic Acids Research.

[62] Zhao X (2015). *YNL134C* from *Saccharomyces cerevisiae* encodes a novel protein with aldehyde reductase activity for detoxification of furfural derived from lignocellulosic biomass. Yeast.

[63] Skoneczna A, Micialkiewicz A, Skoneczny M (2007). Saccharomyces cerevisiae Hsp31p, a stress response protein conferring protection against reactive oxygen species. Free Radical Biology and Medicine.

[64] Caba E, Dickinson DA, Warnes GR, Aubrecht J (2005). Differentiating mechanisms of toxicity using global gene expression analysis in Saccharomyces cerevisiae. Mutation Research/Fundamental and Molecular Mechanisms of Mutagenesis.

[65] Garay-Arroyo A, Covarrubias AA (1999). Three genes whose expression is induced by stress inSaccharomyces cerevisiae. Yeast.

[66] Nordling E, Jörnvall H, Persson B (2002). Medium-chain dehydrogenases/reductases (MDR). Family characterizations including genome comparisons and active site modeling. European journal of biochemistry.

[67] Ansell R (2004). NADH-reductive stress in Saccharomyces cerevisiae induces the expression of the minor isoform of glyceraldehyde-3-phosphate dehydrogenase (TDH1). Current Genetics.

[68] Luttik MA (1998). The Saccharomyces cerevisiae NDE1 and NDE2 genes encode separate mitochondrial NADH dehydrogenases catalyzing the oxidation of cytosolic NADH. The Journal of biological chemistry.

[69] Gömpel-Klein P, Brendel M (1990). Allelism of SNQ1 and ATR1, genes of the yeast Saccharomyces cerevisiae required for controlling sensitivity to 4-nitroquinoline-N-oxide and aminotriazole. Current genetics.

[70] Carlsson M, Hu G-Z, Ronne H (2018). Gene dosage effects in yeast support broader roles for the LOG1, HAM1 and DUT1 genes in detoxification of nucleotide analogues. PloS one.

[71] Lesage G, Bussey H (2006). Cell wall assembly in Saccharomyces cerevisiae. Microbiology and molecular biology reviews: MMBR.

[72] Yu L (2010). Microarray analysis of p-anisaldehyde-induced transcriptome of Saccharomyces cerevisiae. Journal of Industrial Microbiology & Biotechnology.

[73] Soontorngun N (2017). Reprogramming of nonfermentative metabolism by stress-responsive transcription factors in the yeast Saccharomyces cerevisiae. Current Genetics.

[74] Bereketoglu C, Arga KY, Eraslan S, Mertoglu B (2017). Genome reprogramming in *Saccharomyces cerevisiae* upon nonylphenol exposure. Physiological Genomics.

[75] Bergkessel M, Whitworth GB, Guthrie C (2011). Diverse environmental stresses elicit distinct responses at the level of pre-mRNA processing in yeast. RNA (New York, N.Y.).

[76] Ljungdahl PO, Daignan-Fornier B (2012). Regulation of Amino Acid, Nucleotide, and Phosphate Metabolism in *Saccharomyces cerevisiae*. Genetics.

[77] Mara P, Fragiadakis GS, Gkountromichos F, Alexandraki D (2018). The pleiotropic effects of the glutamate dehydrogenase (GDH) pathway in Saccharomyces cerevisiae. Microbial Cell Factories.

[78] Lee YJ, Kim KJ, Kang HY, Kim H-R, Maeng PJ (2012). Involvement of *GDH3* -encoded NADP^+^ -dependent Glutamate Dehydrogenase in Yeast Cell Resistance to Stress-induced Apoptosis in Stationary Phase Cells. Journal of Biological Chemistry.

[79] Grant CM, MacIver FH, Dawes IW (1997). Glutathione synthetase is dispensable for growth under both normal and oxidative stress conditions in the yeast Saccharomyces cerevisiae due to an accumulation of the dipeptide gamma-glutamylcysteine. Molecular Biology of the Cell.

[80] James J (2015). A rapid method to assess reactive oxygen species in yeast using H _2_ DCF-DA. *Analytical*. Methods.

[81] Oliveira R, Johansson B (2012). Quantitative DNA Damage and Repair Measurement with the Yeast Comet Assay. in. Methods in molecular biology (Clifton, N.J.).

[82] Końca K (2003). A cross-platform public domain PC image-analysis program for the comet assay. Mutation research.

[83] Odoni DI (2017). Comparative proteomics of *Rhizopus delemar* ATCC 20344 unravels the role of amino acid catabolism in fumarate accumulation. PeerJ.

[84] Pérez-Rubio P, Lottaz C, Engelmann JC (2019). FastqPuri: high-performance preprocessing of RNA-seq data. BMC Bioinformatics.

[85] Zerbino DR (2018). Ensembl 2018. Nucleic acids research.

[86] Dobin A (2013). STAR: ultrafast universal RNA-seq aligner. Bioinformatics.

[87] Liao Y, Smyth GK, Shi W (2014). featureCounts: an efficient general purpose program for assigning sequence reads to genomic features. Bioinformatics.

[88] Love MI, Huber W, Anders S (2014). Moderated estimation of fold change and dispersion for RNA-seq data with DESeq. 2. Genome Biology.

[89] Maere S, Heymans K, Kuiper M (2005). BiNGO: a Cytoscape plugin to assess overrepresentation of Gene Ontology categories in Biological Networks. Bioinformatics.

[90] Kanehisa M, Sato Y, Kawashima M, Furumichi M, Tanabe M (2016). KEGG as a reference resource for gene and protein annotation. Nucleic Acids Research.

[91] R Development Core Team, R. R: A language and environment for statistical computing. *R Foundation for Statistical Computing, Vienna, Austria* (2013).

[92] Wickham, H. *Ggplot2: elegant graphics for data analysis*. (Springer, 2009).

[93] Cherry JM (2012). Saccharomyces Genome Database: the genomics resource of budding yeast. Nucleic Acids Research.

