# Supplementary material for: Toxicological response of the model fungus Saccharomyces cerevisiae to different concentrations of commercial graphene nanoplatelets
Source: Sci Rep. 2020 Feb 24;10:3232. doi: 10.1038/s41598-020-60101-7 (PMC7039959; doi:10.1038/s41598-020-60101-7)
Supplement: Supplementary file 1 — Supplementary Information. [file 41598_2020_60101_MOESM1_ESM.docx]

Toxicological response of the model fungus *Saccharomyces cerevisiae* to different concentrations of commercial graphene nanoplatelets

Maria Suarez-Diez^1^, Santiago Porras^2^, Felix Laguna-Teno^3^, Peter J. Schaap^1^, Juan A. Tamayo-Ramos^3^*

^1^Laboratory of Systems and Synthetic Biology, Wageningen University & Research, Stippeneg 4 6708WE Wageningen, the Netherlands

^2^Departamento de Economía Aplicada, University of Burgos, Plaza Infanta Doña Elena, s/n, 09001, Burgos, Spain

^3^International Research Centre in Critical Raw Materials-ICCRAM, University of Burgos, Plaza Misael Banuelos s/n, 09001, Burgos, Spain

***Corresponding author:**

Juan Antonio Tamayo-Ramos

Mailing address: International Research Centre in Critical Raw Materials-ICCRAM, University of Burgos, Plaza Misael Banuelos s/n, 09001, Burgos, Spain.

Email: ja.tamayoramos@gmail.com

**Supplementary Table S1: Summary of RNA-Seq mapping**

Summary of RNA-Seq quality analysis and mapping for each of the considered samples. *Raw reads* indicates the total number of reads generated in the sequencing, whereas *Clean reads* indicates the number of reads that passed the quality filtering with FastqPuri. The rest of the columns provide mapping statistics.

**Supplementary Table S2: Differential expression analysis data**

Differential expression analysis of samples exposed to GN concentrations of 160 and 800 mg L^-1^ compared to the control condition (no exposure). Each table contains, for each gene, base mean across samples, log2 fold change, standard error, test statistics, p-value and adjusted p-value of the differential expression, and additional gene annotation. Here, positive log2 fold changes indicate higher expression in the exposure (160 or 800 mg L-1) than in the control condition.

**Supplementary Table S3: Gene Ontology analysis data**

Results of the Gene Ontology enrichment analysis performed with BINGO. Analysis are presented separated for each ontology: biological process (BP), molecular function (MF) and cellular component (CC). Analysis for up and down regulated genes upon exposure to 160 and 800 mg L^-1^ CN are presented separated, as well as analysis on the genes up or down regulated upon both exposures. The first 20 rows provide metadata on each analysis. The results consist on tables that indicate, for each GO identifier (GO-ID): *p-value* of the enrichment, p-value adjusted for multiple testing (*corr p-value*); the number of genes annotated to the selected GO ID in the sample (x) and in the genome (n) and the number of genes annotated to the selected ontology in the sample (X) and in the genome (N)

**Supplementary Table S4: Pathway enrichment analysis data**

Results are presented for up and down regulated genes upon exposure to GN concentrations of 160 and 800 mg L^-1^ compared to the control condition. For each up/down set all results are presented; in a separate sheet (named “_significant”) a selection of pathways with FDR <0.05 in any of the cases is presented. The analysis of the *intersection* set (genes that are differentially expressed upon both exposures) is also presented. Each table contains, for each pathway in KEGG, the number of genes associated to the pathway either in the genome or in the considered sample; the fraction (or ratio) of genes associated to the pathway either in the genome or in the considered sample; the p-value of the enrichment and the false discovery rate (FDR).
